# Supplementary material for: Optimizing sample preparation for culture-free nanopore sequencing to enable rapid pathogen and antimicrobial resistance profiling in bovine mastitis
Source: Front Microbiol. 2025 Nov 6;16:1680165. doi: 10.3389/fmicb.2025.1680165 (PMC12631300; doi:10.3389/fmicb.2025.1680165)
Supplement: Supplementary file 2 [file Table_2.docx]

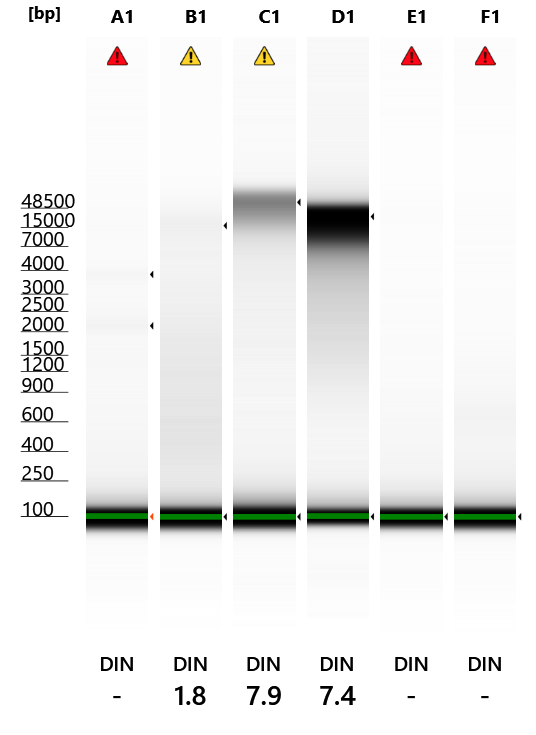

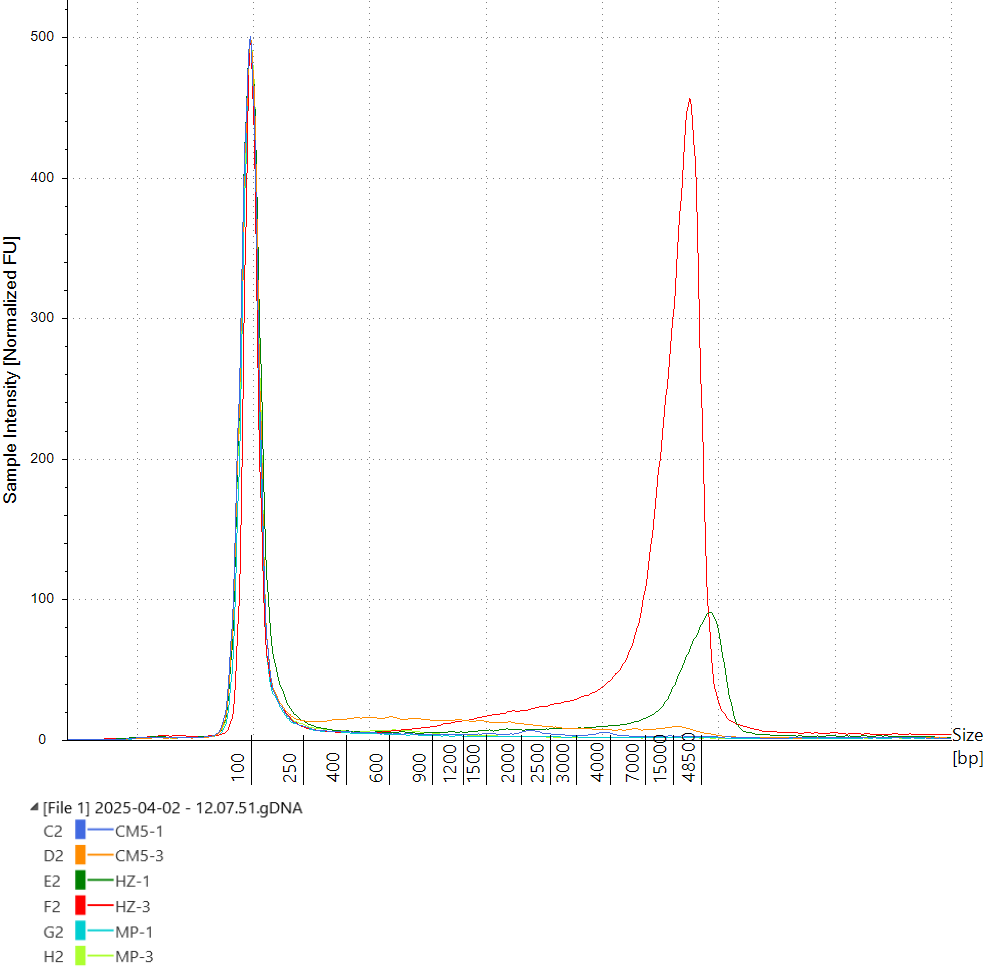


**B**

**A**

**Figure S2.** Assessment of DNA quality and fragment size distribution using Agilent TapeStation system, genomic screen tape. **A.** Sample integrity plot; CM5 : Mol Com5 kit, HZ: Host Zero kit, MP: SPINeasy kit; 1: S. aureus sample, 3: E. coli sample. **B.** Corresponding gel-like image from Agilent TapeStation. Lanes A1 and B1: Mol Com5 S. aureus and E. coli, C1 and D1: HostZero S. aureus and E. coli, E1 and F1: SPINeasy S. aureus and E. coli. Black triangular dots in the lanes represent bands with the highest intensity. HostZero kit has the largest fragment sizes as well as a better DNA integrity (DIN) values. There was no DNA detected in lanes E1 and F1 (DNA extracted with SPINeasy kit).
